# Comparison of Workflows for Milk Lipid Analysis: Phospholipids

**DOI:** 10.3390/foods12010163

**Published:** 2022-12-28

**Authors:** Cheng Li, Zhiqian Liu, Leah Marett, Jennie Pryce, Simone Rochfort

**Affiliations:** 1 Agriculture Victoria Research, AgriBio, 5 Ring Road, Bundoora, VIC 3083, Australia; 2 School of Applied Systems Biology, La Trobe University, Bundoora, VIC 3083, Australia; 3 Agriculture Victoria Research, Ellinbank Centre, Ellinbank, VIC 3821, Australia; 4 Centre for Agricultural Innovation, School of Agriculture and Food, Faculty of Veterinary and Agricultural Sciences, The University of Melbourne, Melbourne, VIC 3010, Australia

**Keywords:** milk, phospholipids, quantification, liquid chromatography-mass spectrometry

## Abstract

Milk is a rich source of lipids, with the major components being triglycerides (TAG) and phospholipids (mainly phosphatidylcholine (PC), sphingomyelin (SM), phosphatidylethanolamine (PE), phosphatidylserine (PS) and phosphatidylinositol (PI)). Liquid chromatography-mass spectrometry (LC-MS) is the predominant technique for lipid identification and quantification across all biological samples. While fatty acid (FA) composition of the major lipid classes of milk can be readily determined using tandem MS, elucidating the regio-distribution and double bond position of the FA remains difficult. Various workflows have been reported on the quantification of lipid species in biological samples in the past 20 years, but no standard or consensus methods are currently available for the quantification of milk phospholipids. This study will examine the influence of several common factors in lipid analysis workflow (including lipid extraction protocols, LC stationary phases, mobile phase buffers, gradient elution programmes, mass analyser resolution and isotope correction) on the quantification outcome of bovine milk phospholipids. The pros and cons of the current LC-MS methods as well as the critical problems to be solved will also be discussed.

## 1. Introduction

Bovine milk contains 3–5% of fat, making it one of the major components of milk solids alongside lactose and proteins. Milk fat is present as microscopic globules with triacylglycerols (TAG) as the core content and tri-layer phospholipids as the globule membrane. While TAG is considered mainly an energy source, phospholipids including mainly phosphatidylcholine (PC), phosphatidylethanolamine (PE), sphingomyelin (SM), phosphatidylserine (PS) and phosphatidylinositol (PI) are bioactive molecules, offering multiple benefits to human health [1,2,3]. As a result, there is a growing interest in using milk fat globule membrane material as an ingredient of infant formulas [4]. In addition, some phospholipids of milk were found to be potential biomarkers for the health status of dairy cows [5]. Consequently, accurate quantification of phospholipids in milk, isolated milk fat globule membrane and other dairy products is gaining increasing attention [6,7].

Several analytical systems have been reported for milk phospholipid measurement, the most common ones being LC-ELSD, shotgun-MS, LC-MS and ^31^P-NMR. Normal-phase LC-ELSD/Hilic-ELSD has been used for milk phospholipid analysis for over two decades (for review: see Liu et al. [8]). It provides separation of the major phospholipid classes of milk (PC, PE, SM, PS and PI), and the ELSD response is thought to be independent of fatty acid (FA) structures of the lipids [9]. This is a clear advantage over an MS detector. However, the drawbacks of this detector include: (1) no species information can be obtained; (2) it is prone to interference by co-eluting analytes as ELSD is a universal detector; (3) it has lower sensitivity as compared to MS. 

Several studies have been reported recently on the use of ^31^P-NMR for phospholipid quantification in milk and dairy products [10,11,12]. Although the sample preparation for NMR is simple and quantification of phospholipids based on ^31^P signal is reliable, the intrinsic low sensitivity of NMR remains the main obstacle for its widespread use. Despite the perceived shortcomings of shotgun-MS (especially that of ion suppression for low-abundance lipid species), the direct-infuse-MS method has been proven to be valuable for high throughput lipid profiling and is being frequently used in lipidomic analysis [13,14,15,16,17,18,19].

The most widely used technique in lipidomic analysis of biological samples including milk is indisputably LC-MS (for reviews: see [8,20,21]. For example, RP-LC-MS has been a popular choice in global lipidomic profiling of human plasma of large cohorts, enabling several hundred lipid species (polar and non-polar) to be quantified in a single analysis [22]. Hilic-MS is also suitable for phospholipid analysis and has been applied to milk phospholipid profiling in numerous studies [23,24,25,26,27]. Like normal-phase-LC, Hilic separates different polar lipid classes based on their head group but provides minimal separation of species within each class. 

For both RP-LC-MS and Hilic-MS, the variation of ESI-MS response with FA structure is the major challenge for lipid quantification in milk and other biological samples [18,21,28], due to the huge number of lipid species present in milk and very few lipid standards being commercially available. Using Hilic-QqQ-MS, Fong et al. [29] demonstrated that the MS response is mainly determined by the head group whereas the FA structure has little influence when the concentration of phospholipids is below a certain level. This finding is significant and is a strong justification for Hilic-MS-based polar lipid quantification using one standard for each class [26,27,30]. Despite being not fully validated, this one-standard quantification approach has also been widely adopted in RP-LC-MS-based polar lipid and TAG analysis [31,32,33,34]. This has led to the commercialisation of some proprietary lipidomic standard mix containing one deuterated standard per lipid class by Avanti Lipids. Although reliable for relative quantification (or fold-change comparison between sample groups), the utility of using one single standard for absolute quantification of lipids by RP-LC-MS remains unclear.

When using ESI-MS, the ionization efficiency (or response) of lipids is expected to be influenced by mobile phase composition, additives and sample matrix. While mobile phase composition at a specific retention time (RT) is determined by the elution gradient and the most frequently used mobile phase additives include ammonium formate and ammonium acetate, the sample matrix is in turn related to the lipid extraction methods (e.g., Folch method vs. one-phase method). How each of these factors influences the lipid quantification results remains to be investigated.

Compared to small molecules encountered in metabolomics study, lipids are complex molecules containing a large number of carbon and hydrogen atoms, so the contribution and interference of C13 isotope in particular is expected to be significant for both identification and quantification [35]. However, until now, few researchers have considered this in lipidomic analysis [18,19,36,37]. Clearly, both type 1 isotope (monoisotopic proportion) and type 2 isotope (mainly M+2) corrections are relevant to milk lipid quantification, but whether both corrections are necessary for the quantification of phospholipids and TAG remains to be clarified.

In this study, we have systematically evaluated several key factors including lipid extraction method, LC stationary phase, mobile phase buffer, LC elution gradient, MS resolution settings as well as type 1 and type 2 isotope corrections for their effect on milk phospholipid quantification at the species and class levels. The five major phospholipid classes (PC, PE, SM, PS and PI) were measured in this study. 

## 2. Materials and Methods

### 2.1. Milk Source and Chemicals

Two full cream commercial milk samples (named Milk-11 and Milk-12) obtained from a local supermarket (Victoria, Australia) were used in this study; the total fat content was 3.4% for both samples as shown on the label. 

Mouse Splash^®^ Lipidomix standards (a mix of 14 deuterated standards, see Table S1 for details), were purchased from Avanti Lipids (Alabaster, AL, USA). Solvents used for milk lipid extraction and mobile phase preparation were of chromatographic grade and were from Merck (Rahway, NJ, USA) (methanol, butanol and acetonitrile) and Sigma-Aldrich (St. Louis, MO, USA) (chloroform and isopropanol). Ammonium formate and ammonium acetate, used as mobile phase additive, were of LC-MS grade (Sigma-Aldrich).

### 2.2. Lipid Standard Preparation and Lipid Extraction from Milk Samples

Mouse Splash Lipidomix standard mix were diluted by adding 1 mL of methanol and stored at −20 °C. Working solutions of Mouse Splash standard mix were prepared by diluting the stock solution 32-, 16-, 8- and 4-fold with a solvent mix (butanol/methanol/chloroform, 3:5:4, *v/v/v*). 

Two protocols for lipid extraction were compared in this study, namely the Folch method [38] and the one-phase method [39]. For the Folch method, 1 mL of milk, 1 mL of water and 8 mL of chloroform/methanol (2:1, *v/v*) were mixed at room temperature in a 15-mL centrifuge tube; lipids were extracted by vortex at full speed for 2 min, followed by centrifugation for 15 min (at 5500× *g*) to separate the aqueous and organic phases. After removal of the organic phase, the aqueous phase was extracted again with 4 mL of chloroform/methanol (2:1, *v/v*). The organic phases were then combined and dried at 30 °C under N2 to remove the solvents. The residual lipids were then redissolved in 2 mL of the solvent mix (butanol/methanol/chloroform, 3:5:4, *v/v/v*) and stored at −20 °C before LC-MS analysis. For the one-phase method, 100 µL of milk was mixed with 1 mL of butanol/methanol/chloroform (3:5:4, *v/v/v*) in a 2-mL eppendorf tube, and this mixture was shaken vigorously by vortex for 30 s and then centrifuged for 10 min (at 13,000× *g*). The supernatant was used directly for lipid analysis by LC-MS. 

### 2.3. Phospholipid Analysis

Phospholipids of milk were analysed by RP-LC-MS and Hilic-MS. For RP-LC-MS, phospholipids were separated by an Eclipse XDB-C8 column (150 × 2.1 mm, 3.5 µm, Agilent Technologies (Santa Clara, CA, USA)) on a Vanquish UHPLC system (Thermo Fisher Scientific (Waltham, MA, USA)). The column compartment was maintained at 55 °C and the sample tray at 12 °C. The mobile phase is composed of 60% acetonitrile containing 10 mM ammonium formate or 10 mM ammonium acetate (A) and acetonitrile/isopropanol (10:90, *v/v*) containing 10 mM ammonium formate or 10 mM ammonium acetate (B). Two gradient elution programmes were compared, namely multistep gradient as reported by Hu et al. [40] and linear gradient (a linear increase of mobile phase B from 5 to 100% over 25 min with a total runtime of 34 min). The flowrate was 0.25 mL/min for both programmes and the injection volume was 4 µL.

In the case of Hilic-MS, a Kinetex Hilic column (100 × 2.1 mm, 1.7 µm, Phenomenex (Torrance, CA, USA)) was used for lipid separation. The column compartment was maintained at 30 °C and the sample tray at 12 °C. The mobile phase is composed of 10 mM ammonium formate (A) and acetonitrile containing 0.1% formic acid (B). Three gradient elution programmes were compared, namely Hilic-M4, Hilic-M5 and Hilic-M6 (Table S2). The flowrate was 0.3 mL/min for all programmes and the injection volume was 3 µL.

### 2.4. Mass Spectrometry Settings 

An LTQ-Orbitrap Elite mass spectrometer (Thermo Fisher Scientific) equipped with a heated electrospray ionisation (HESI) source was used for the detection of all lipid classes. The heated capillary was maintained at 300 °C with a source heater temperature of 300 °C, and the sheath, auxiliary and sweep gases were, respectively at 30, 10 and 0 units. The instrument was operated in positive (4.2 kV) ion mode with a full scan (120–1500 *m*/*z*) at a resolution of 60,000 or 240,000. Where MS2 scans were acquired for lipid structural determination, a precursor isolation width of 1 Da, a collision energy of 35 eV (CID mode) and a dynamic exclusion of 5 s were selected. 

### 2.5. Quantification of Phospholipids 

The one-point internal standard (IS) calibration method was applied to phospholipid quantification. Peak area of each lipid species was extracted from the MS spectra using Xcalibur software and the accurate monoisotopic mass (M+H for PC, SM, PE and PS, M+NH4 for PI). The concentration of a lipid species = (peak area of the lipid species/peak area of the IS) × concentration of the IS. Where necessary, the content of each lipid class is estimated by the sum of all species measured within the same class. 

### 2.6. Statistical Analysis

All experiments were performed with 2–4 technical replicates. The results were subjected to ANOVA or Student’s *t*-test (Excel, Microsoft 365) depending on the number of treatments. All experiments were repeated at least twice to confirm the findings. 

## 3. Results

### 3.1. Analysis of Milk Phospholipids by RP-LC-MS

#### 3.1.1. Ammonium Acetate vs. Ammonium Formate as Mobile Phase Additive

**Figure 1:**
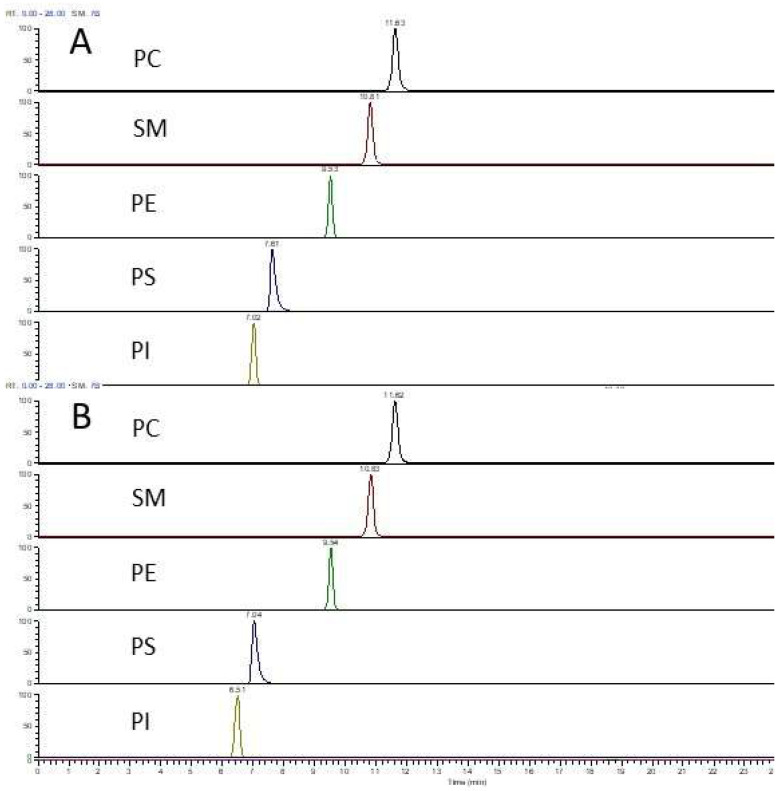
RP-LC-MS profile of phospholipid standards (Mouse Splash Lipidomix standards diluted by 32-fold). The structural information on these deuterated standards is given in Table S1. LC separation was by the Eclipse XDB-C8 column using the multistep gradient elution. Mobile phase additive was 10 mM ammonium formate (**A**) or 10 mM ammonium acetate (**B**) in both binary components.

Both ammonium formate and ammonium acetate are widely used as volatile buffer in RP-LC-MS-based lipidomic analysis. Using the Mouse Splash Lipidomix standards we found both additives attained similar and satisfactory peak shapes for phospholipids and the RT was also very close for PC, SM and PE; a slightly shorter RT was observed for PS and PI with ammonium acetate (Figure 1A,B). 

**Figure 2:**
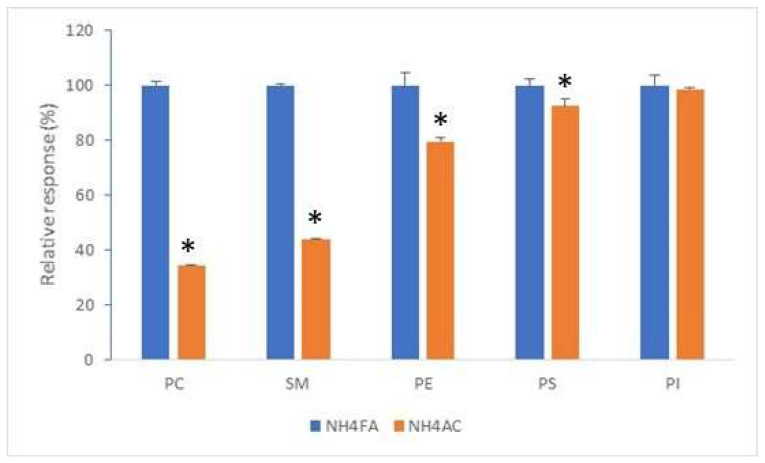
Relative response of phospholipid standards (Mouse Splash Lipidomix standards diluted by 32-fold) as influenced by mobile phase additives. LC separation was by the Eclipse XDB-C8 column using the multistep gradient elution. Mobile phase additive was 10 mM ammonium formate or 10 mM ammonium acetate in both binary components. Each column is the mean of 3 analyses (±SD). * Indicates significant difference by *t*-test (*p* < 0.05).

The ESI-MS response varied with both lipid classes and additives. Although the varying concentration of individual lipids in the Mouse Splash Lipidomix standards make it difficult to determine limit of detection for each lipid class, the peak area (relative response) of these lipid standards (diluted by 32-fold) differed substantially between these two ammonium salts, with ammonium formate giving a much higher response for PC, PE and SM and a slightly higher response for PI and PS as compared to ammonium acetate, under the same LC and MS settings (Figure 2). Similar response patterns were observed with milk endogenous lipids (results not shown). This implies that ammonium formate confers a higher sensitivity in detecting most of the phospholipid classes, a feature that is valuable when dealing with low abundance lipid species. Therefore, ammonium formate was used in all the subsequent RP-LC-MS analyses presented in this report.

#### 3.1.2. One-Phase Extraction vs. the Folch Method

Although validated in our previous study [39], the one-phase method for lipid extraction from milk has not found wide-spread adoption, presumably due to the lack of systematic evaluation on its performance with regard to milk lipid quantification. The lipid extraction efficiency and matrix effect on lipid detection of this one-phase method was further compared against the Folch method. 

**Figure 3:**
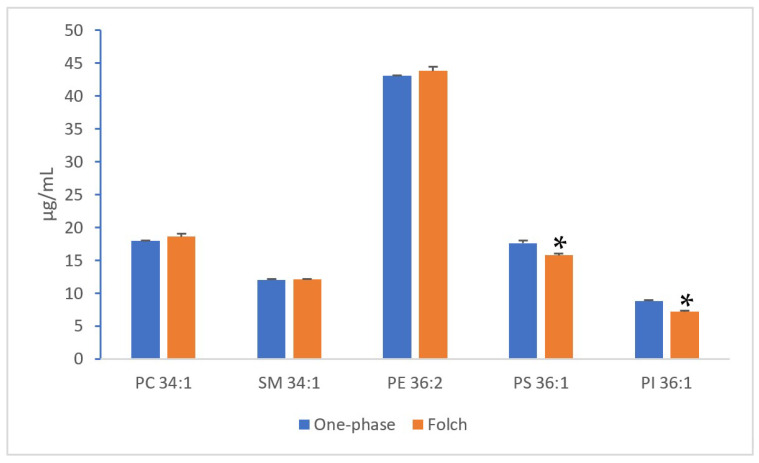
Yield of some phospholipid species (from Milk-11) as influenced by the extraction methods. Sample dilution factor (final analysed sample volume vs. the starting milk volume) was 44-fold and 40-fold for the one-phase method and the Folch method, respectively. LC separation was by the Eclipse XDB-C8 column using the multistep gradient elution. Lipid concentration was measured using the one-point IS calibration method. Each column is the mean of 3 analyses (±SD). * Indicates significant difference by *t*-test (*p* < 0.05).

In the first instance, the yield of one abundant species from each of the PC, SM, PE, PS and PI classes of one milk sample (Milk-11) was compared. It was found that the concentration of the PC, SM and PE species was similar between the two extraction protocols, but the one-phase method was superior in extracting PS and PI from milk (Figure 3). 

#### 3.1.3. Matrix Effects 

The matrix effect (%) was estimated by the response (peak area) ratio of deuterated standards in the presence of matrix (lipid extract) to that without matrix (in pure solvents). As matrix effect is affected by both lipid extraction method (one-phase matrix being different from Folch matrix) and LC separation methods. Two extraction methods and two RP-LC elution programmes were compared using two milk samples (Milk-11 and Milk-12).

For all phospholipids, the matrix effect ranged from 82 to 116% (in most cases from 89 to 112%), implying no remarkable ion suppression or ion enhancement was detected in the presence of milk lipophilic fraction (isolated by the Folch method) or the deproteinised milk matrix (derived from the one-phase method) for both samples, regardless of LC elution regime (Table 1). Interestingly, a slightly greater ion suppression was found to be associated with the linear gradient as compared to the multistep gradient in the case of PC (82–90% vs. 89–95%). 

#### 3.1.4. Phospholipid Quantification as Influenced by LC Elution Programmes

While no significant matrix effect was observed for phospholipid standards using either LC elution programmes, the effect of LC elution programme on the quantification of phospholipid species using one single IS remained unknown. The concentration of the major species of PC and PE as well as the sum concentration of each class was compared across the two LC elution regimes. 

Table 2 shows that a significant difference was found both at the individual PC species level (5 out of the 8 species) and the total PC class level between the two LC elution programmes. In the case of the PE class, a significant difference was found for one out of the 7 species and also for the sum concentration (Table 2). For both PC and PE classes, the sum concentration determined by the multistep gradient method was around 10% higher than that obtained by the linear gradient method. In addition, the overall peak shape of milk phospholipids appears to be better with the multistep gradient (results not shown).

#### 3.1.5. Type 1 Isotopic Correction Effect

C8 stationary phase can readily resolve analogous phospholipid species differing in one double bond. Consequently, the type 2 (or M+2) isotopic correction is not necessary when using RP-LC-MS, regardless of the resolving power of the mass analyser used. 

When using one IS for phospholipid species quantification, the type 1 isotope correction becomes relevant, although the mass range of the major species is much narrower (PC: 700–800; SM: 700–820; PE: 690–750; PS: 760–840; PI: 850–910 Da) and the correction factor for these species is much closer to that of the IS contained in the Mouse Lipidomix standards (Table 3). As a result, the type 1 isotopic correction is expected to provide only a modest adjustment to the original results for PC and PE species (96.7 to 103.4% for PC and 98.9 to 103.4% for PE), but a non-negligible change (95.6 to 107%) for SM species as compared to non-corrected results. 

### 3.2. Analysis of Milk Phospholipids by Hilic-MS

#### 3.2.1. One-Phase Extraction vs. the Folch Method

For all phospholipids, the matrix effect ranged from 78 to 104% for the Folch extract and from 90–117% for the one-phase matrix, suggesting only a modest matrix effect (ion suppression and ion enhancement) was detected with Hilic-MS, when the lipid extract was appropriately diluted (40- and 44-fold for Folch and one-phase method, respectively) (Table 4). These dilution factors were found to be adequate for reliable quantification of all major phospholipid species. It is worth noting that SM and PS tend to see a slight ion enhancement with the one-phase extract and the Folch extract tends to show a higher ion suppression, whereas no difference was found between the two milk samples. 

As discussed with RP-LC-MS, the performance of the one-phase and the Folch methods in lipid quantification using Hilic-MS was compared by measuring the concentration of one abundant species from each of the PC, SM, PE, PS and PI classes of the two milk samples (Milk-11 and Milk-12). Both extraction methods generated similar results for PC, SM and PE species but the one-phase method tended to show a higher extraction efficiency for PS and PI (Figure S1). 

#### 3.2.2. Type 1 Isotope Correction

The type 1 isotope correction is related mainly to the carbon number difference between the analytes and the IS and is independent to the LC separation mode. As the implication of type 1 isotope correction for phospholipid quantification using the Mouse Lipidomix standards was already discussed previously in the RP-LC-MS section (Section 3.1.5), the same rules can be applied to Hilic-MS.

#### 3.2.3. Type 2 Isotope Correction (M+2 Isotope Interference)

Unlike with RP-LC-MS, where all analogous species differing by one double bond can be chromatographically separated, making type 2 correction irrelevant, Hilic can only separates phospholipid classes based on their head groups, so all species of the same class essentially co-elute. In such a case, M+2 isotope may interfere with both identification and quantification of phospholipids at the species level. 

**Figure 4:**
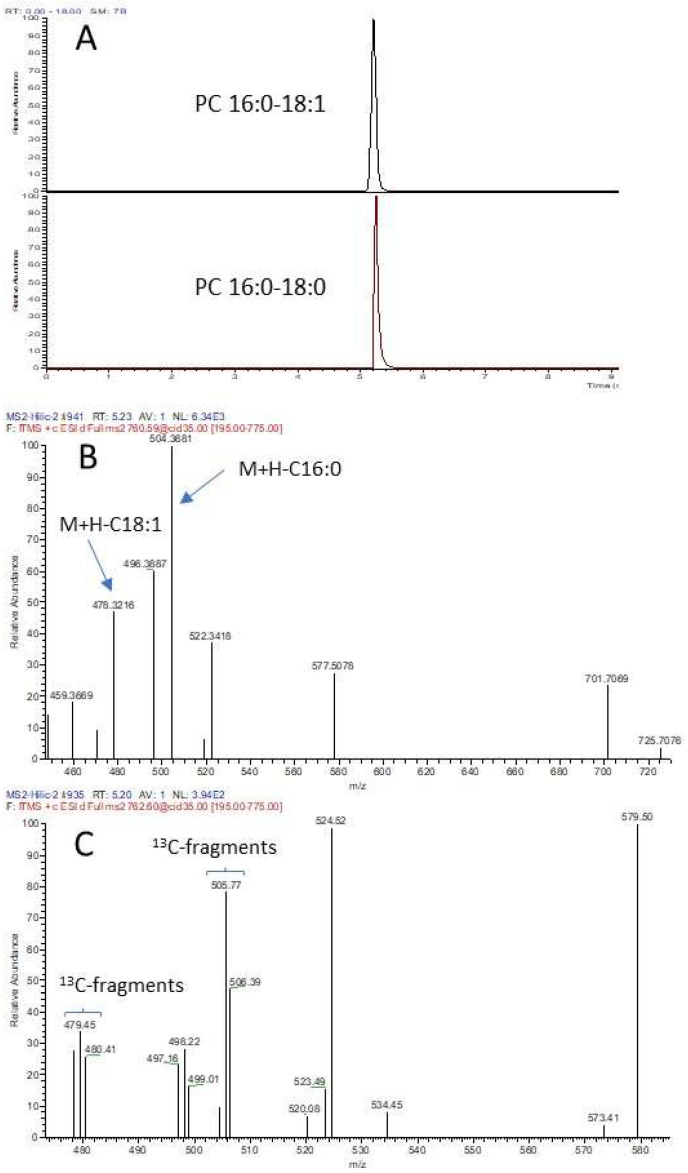
Hilic-MS profile of PC 34:1 and PC 34:0 parent ions and their respective MS2 spectra. Milk lipids were extracted using the Folch method and LC separation was by Hilic-4 method. (**A**): EIC of parent ions of PC 34:1 and PC 34:0; (**B**): MS2 spectrum of PC 34:1; (**C**): MS2 spectrum of PC 34:0.

Figure 4 illustrates how M+2 isotope hinders the identification of lipid species. The coelution of two analogous species PC 34:1 and PC 34:0 is shown in Figure 4A and their respective MS2 spectra in Figure 4B,C. While the MS2 spectrum of PC 34:1 is simple with fragment ions (*m*/*z* 478 and 504) indicating the identity of the two FA (C18:1 and C16:0), that of PC 34:0 is more complex. Indeed, the parent ions of M+2 isotope of PC 34:1 and that of PC 34:0 have a mass difference of 0.0089 Da, making it impossible to isolate either one for fragmentation. Consequently, both ions were fragmented to generate a hybrid MS2 spectrum (Figure 4C), which is characterized by the fragment ion clusters derived from random segregation of ^13^C of the precursor. Clearly, the detection of such ion clusters is an indicator of an M+2 isotope being fragmented. However, the presence of PC 34:0 cannot be confirmed based solely on the hybrid MS2 spectrum. This example shows the limitations of using Hilic-MS for lipid species-level identification, due to the co-elution of isobaric ions, which are widespread in all classes of milk lipids. 

**Figure 5:**
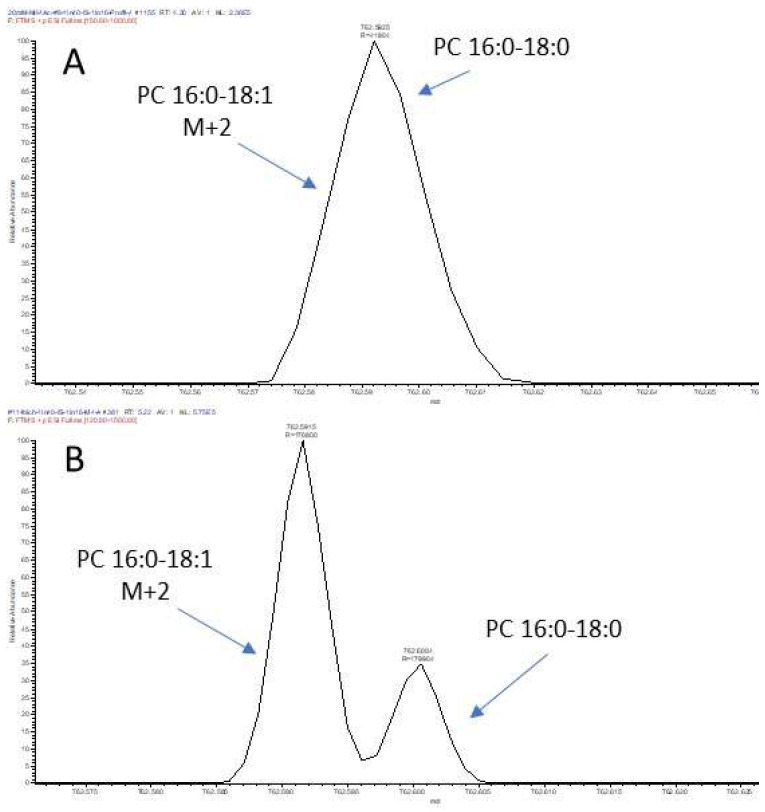
Mass separation of M+2 isotope of PC 34:1 (*m*/*z* 762.5923) and monoisotopic ion of PC 34:0 (*m*/*z* 762.6013) by Orbitrap Elite at two resolution settings. (**A**): 60,000; (**B**): 240,000.

The presence of coeluting M+2 isotope could also interfere with the quantification of phospholipid species. Figure 5 shows that when Orbitrap Elite mass analyzer was operated at a resolution of 60,000, the M+2 isotope of PC 34:1 cannot be resolved from the monoisotopic ion of PC 34:0 (Figure 5A). In this case, the type 2 isotope correction becomes necessary for accurate quantification of the latter. However, when the same mass analyzer was set to the maximum resolution of 240,000, a quasi-total separation was observed for these two isobaric ions (Figure 5B), despite the actual resolution achieved at *m*/*z* 762 was around 170,000. This suggests that the type 2 isotopic correction is not necessary only when using a mass analyzer enabling a resolving power equal to or above 170,000 at the actual isobaric species (*m*/*z*) level.

#### 3.2.4. Elution Programme Comparison

The potential LC elution programmes on the measurement of phospholipids of milk was evaluated by comparing the concentration of individual species and the sum concentration of the five classes.

**Figure 6:**
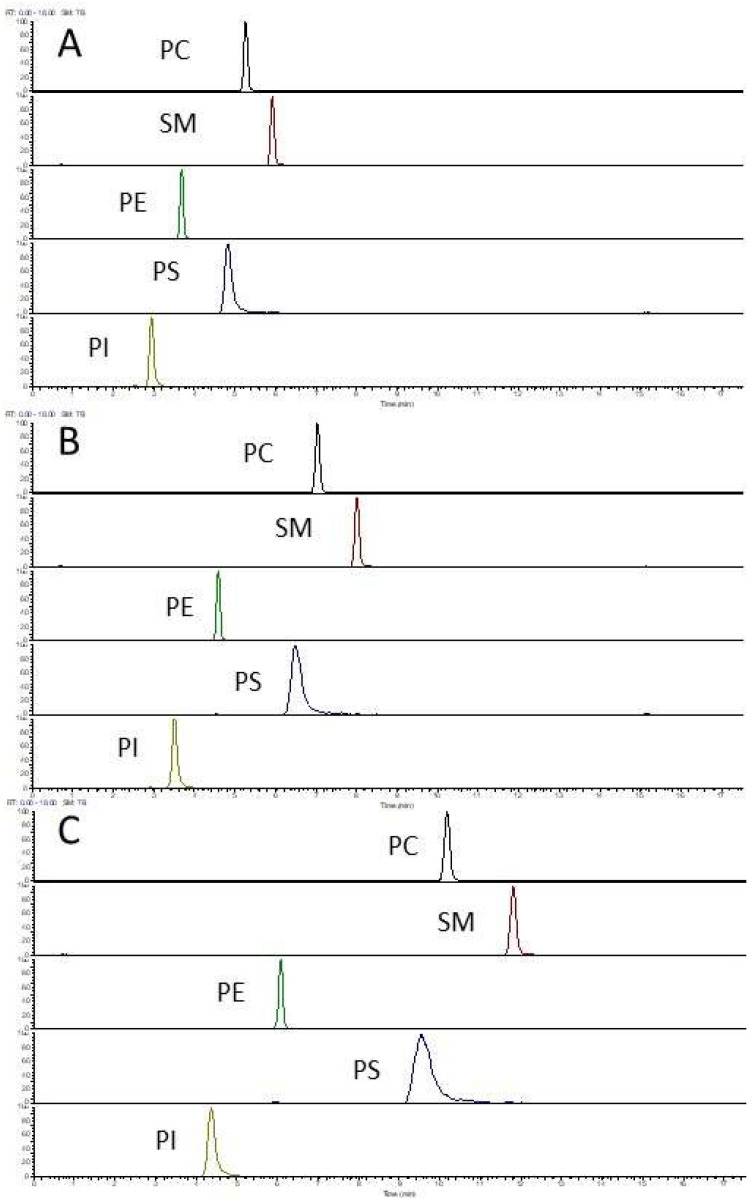
Hilic-MS profile of phospholipid standards. LC separation was by the Kinetex Hilic column using three elution programmes ((**A**): Hilic-M4; (**B**): Hilic-M5 and (**C**): Hilic-M6). The standards used were Mouse Splash Lipidomix standards (diluted by 32-fold).

Both the retention time and the peak shape were influenced by the elution programmes. While satisfactory peak shape was obtained for PC, SM, PE and PI regardless of elution gradients, peak broadening was observed for PS when a shallower gradient was applied (Figure 6). 

Different elution programmes also affected the MS response of phospholipids as expected (results not shown). However, the relative proportion of individual species within each class as well as the sum concentration of each class are very similar across the three LC elution methods (Table 5). Considering the overall peak shape for all phospholipid classes and the total runtime, Hilic-M4 is a preferred LC elution method.

#### 3.2.5. Comparison of RP-LC-MS and Hilic-MS for the Quantification of Phospholipids

Both RP-LC-MS and Hilic-MS can be used to quantify phospholipids. In this study, PC, SM and PE concentration of milk determined by C8-LC-MS and Hilic-MS at the species level and the sum concentration levels was compared. Table 6 shows that PC species tend to be detected at a higher concentration with Hilic-MS, whereas the opposite was found with SM species. In the case of PE class, no consistent trend was observed across different species, although the sum concentration was higher with RP-LC-MS. 

While all species within the same class essentially coeluted with Hilic separation, the retention time scattered substantially with RP separation as compared to the IS. This may affect the ionization efficiency of different species within the same class as compared to the IS, leading to variable measurement results. The retention time of the major species and that of the IS for PC, SM and PE classes is shown in Figure S2.

## 4. Discussion

Quantification of lipids especially phospholipids in milk and dairy products are gaining increasing interest for their perceived beneficial effects on human health. While normal-phase-LC coupled to ELSD detector is the classic method for class-level phospholipid quantification, ^31^P-NMR is gaining popularity for its simplicity in sample preparation and reliability in quantification. However, for species-level quantification, LC-MS is still the preferred option for most researchers. 

While numerous studies have been devoted to comprehensive identification of milk lipid molecular species, accurate quantification of lipids at the species level is currently unachievable. Three reasons are responsible for this. (1) A large number of isomer species have been identified in milk. (2) Chromatographic separation of all these isomers is currently unfeasible regardless of LC column type, length and elution gradient. Indeed, a multi-dimensional chromatography system may be needed to achieve this goal. (3) The number of lipid standards is very limited as compared to the thousands of species identified in milk. Consequently, until now lipid quantification with milk samples has been mostly performed at the sum structure level (i.e., the sum of all isomers having the same molecular mass and the same chemical formula) for both TAG and polar lipids. 

Numerous workflows have been reported for lipidomic analysis of biological samples in the past 20 years. The large number of options concerning lipid extraction, LC separation, MS detection and quantification methods are overwhelming and a standard or consensus workflow for milk lipid quantification is lacking. To address this, we undertook a systematic evaluation of the current procedures for quantification of the major lipid classes in bovine milk. 

The Folch method is still the most popular protocol for lipid extraction from milk, although the one-phase method we reported several years ago is much simpler [39]. In this study the one-phase method was found to be associated with a slight ion enhancement for SM and PS regardless of LC methods. It is known that the matrix derived from one-phase extraction is more complex than the Folch matrix, but the underlying mechanisms for the ion enhancement remain to be investigated. Overall, the one-phase method is as suitable as the Folch method for lipid quantification either by RP-LC-MS or by Hilic-MS. In addition, the higher efficiency of the one-phase method in extracting PS and PI from milk (as compared to the Folch method) has been confirmed. As a result, the application of the one-phase method is encouraged for LC-MS-based milk lipidomic analysis, especially for the processing of large number of samples. 

Regarding the mobile phase additives, both ammonium formate and ammonium aetate have been widely used in lipidomic analysis workflows [41,42]. Our study revealed that the former offers a much higher MS response for most lipid classes, hence a better sensitivity than the latter. Although the underlying mechanism for the differential ionization efficiency of lipids in the presence of these ammonium salts is unknown, this is the first report showing the advantage of using ammonium formate as mobile phase buffer in lipid analysis by LC-MS.

Quantification of lipids is often carried out with one standard per lipid class due to the limited availability of standards [27,43]. Although either the one-point IS calibration method or one-standard external calibration method is useful for relative comparison or for determination of fold-change between sample groups, the reliability of such approaches for accurate quantification of lipids at the species- or class-level requires a thorough investigation. 

Like C18, C8 provide species-level separation, facilitating lipid identification. However, such a separation also means that different species elute at different retention times, some close to while others distant from the IS. The ionization efficiency of phospholipids is primarily determined by the head group. However, retention times, which are related to mobile phase composition, could also affect the ionization of analytes. Consequently, the response factors are expected to be different for different species of the same lipid class when using RP-LC-MS. In such a case, the accuracy of quantification using one single standard is questionable. The fact that inconsistent results were obtained with two RP-LC elution programmes is a direct proof for this. To compensate for the response variation across the spectrum of a lipid class, multiple standards were used in the quantification of SM species [19]. 

Adding IS prior to lipid extraction is the best way to correct any errors related to extraction efficiency, solvent evaporation, inject volume variation and detection fluctuation. Unfortunately, in the case of milk lipids, only deuterated standards are suitable to be spiked into the samples, as endogenous lipids are present for any molecular mass. Given the high cost of deuterated lipid standards, adding them to each sample will significantly increase the analytical cost. Through the post-extraction IS spiking test, we have found the matrix effect is modest when the milk matrices are appropriately diluted, suggesting that the external calibration method (with deuterated or non-labelled standards) can be used for quantification to reduce the sample processing cost. In such a case, the signal variation caused by the injection volume inaccuracy and/or detector fluctuation can be offset by performing replicate analyses, given the extraction efficiency by the Folch and the one-phase method has been well proven.

For polar lipid analysis, in addition to RP-LC-MS, Hilic-MS is widely used [8]. Although the coelution of all species within the same class hinders the identification caused by M+2 isotope interference, the overall peak shape is clearly better than RP-LC and the quantification results are more consistent regardless of elution gradients. Owing to its robustness and high compatibility with ESI-MS, Hilic-MS has become a popular technique for polar lipid analysis of milk. 

The type 1 and/or type 2 isotope corrections are rarely applied in milk lipid analysis. We have shown in this study that type 1 correction may be necessary when using one or a few standards for each lipid class. As for the type 2 correction, it is essential for Hilic-MS-based lipidomic analysis unless a high-resolution mass analyser is used, as the mass difference of M+2 isotope of one species with the mono-isotope of another one is about 0.009 Da. According to Bielow et al. [15], a mass analyser with a resolving power of around 170,000 is required to resolve such a mass distance for ions with *m*/*z* around 760. When used at a resolution of 240,000, Orbitrap Elite can achieve an actual resolution between 170,000 and 175,000 at *m*/*z* 760, thus allowing suitable separation of M+2 isotopes of phospholipid species of milk. Alternatively, a lower-resolution MS (≤60,000) is unable to resolve M+2 isotope from the adjacent species, which could lead to overestimation of several-fold of the latter depending on the relative abundance of the pair if type 2 correction is not performed.

When RP-LC- and Hilic-MS are compared for quantifying phospholipids of the same milk samples, a large difference was observed for the relative proportion of lipid species as well as the sum concentration of PC, SM and PE classes, highlighting the complexity of accurate quantification of lipids using LC-MS. Based on our findings, for phospholipids, we recommend a workflow combining one-phase extraction with Hilic separation, one-point IS calibration and a high-resolution MS (or low-resolution MS plus type 2 isotope correction).

In conclusion, various key steps in lipid quantification protocols have been compared in this study. While the reliability of the one-phase method for lipid extraction from milk has been confirmed, a significant variation in lipid quantification results was observed between RP-LC-MS and Hilic-MS and even between RP-LC elution programmes. In addition, we have demonstrated the importance of type 1 and type 2 isotopic corrections in lipid quantification. Our study highlights the urgent need for a comprehensive LC-MS and ^31^P-NMR cross-validation for milk phospholipid quantification and calls for the production and commercialisation of reference milk (or milk powder) samples and a deuterated lipid standard mix suitable for milk lipid analysis.

## Figures and Tables

**Table 1:** Matrix effect (%) in relation to lipid extraction methods and LC elution gradients.

Lipid	Milk-11				Milk-12			
	Linear		Multistep		Linear		Multistep	
	One-Phase	Folch	One-Phase	Folch	One-Phase	Folch	One-Phase	Folch
PC	89 ± 2.7	84 ± 3.7	95 ± 0.3	92 ± 4.3	90 ± 2.4	82 ± 3.4	95 ± 2.5	89 ± 3.7
SM	109 ± 1.3	102 ± 3.7	112 ± 3.2	108 ± 4.1	109 ± 3.5	98 ± 3.5	111 ± 1.8	103 ± 5.3
PE	112 ± 1.6	102 ± 4.2	112 ± 3.5	106 ± 5.7	110 ± 4.7	99 ± 2.4	108 ± 0.6	106 ± 4.3
PS	112 ± 7.8	95 ± 4.0	116 ± 1.4	100 ± 3.8	110 ± 6.2	95 ± 7.4	109 ± 0.9	104 ± 4.7
PI	105 ± 5.5	95 ± 5.5	103 ± 1.6	93 ± 6.0	105 ± 0	94 ± 10.4	102 ± 1.6	96 ± 10.9

Note: The standards used were Mouse Splash Lipidomix standards (diluted by 32-fold). The standards were spiked into a 44-fold diluted one-phase matrix or a 40-fold diluted Folch matrix. Each value is the mean of two analyses (±SD).

**Table 2:** Concentration (µg/mL) of the major PC and PE species as determined by the two LC elution methods.

Major Species	Multistep Gradient	Linear Gradient
PC 30:0	8.76 ± 0	9.34 ± 0.03 *
PC 32:1	4.42 ± 0.05	5.00 ± 0.12 *
PC 32:0	10.39 ± 0.27	9.07 ± 0.15 *
PC 34:2	5.31 ± 0.03	5.29 ± 0.01
PC 34:1	18.54 ± 0.46	16.98 ± 0.49
PC 36:3	3.74 ± 0.02	3.72 ± 0.09
PC 36:2	7.73 ± 0.05	6.80 ± 0.25 *
PC 36:1	4.26 ± 0.05	2.48 ± 0.06 *
PC sum	63.16 ± 0.73	58.68 ± 0.72 *
PE 32:1	1.53 ± 0.07	1.35 ± 0.06
PE 34:2	6.68 ± 0.24	6.61 ± 0.08
PE 34:1	13.35 ± 0.24	12.89 ± 0.44
PE 36:4	3.55 ± 0.05	3.44 ± 0.05
PE 36:3	14.54 ± 0.31	14.24 ± 0.11
PE 36:2	43.69 ± 0.56	40.77 ± 0.93
PE 36:1	20.87 ± 0.05	16.26 ± 0.01 *
PE sum	104.19 ± 1.28	95.56 ± 1.68 *

Note: Lipids were extracted by the Folch method. Sample dilution factor was 40. The concentration of lipid species was measured using the one-point IS method. Each value is the mean of 3 analyses (±SD). * Indicates significant difference (*p* < 0.05) by *t*-test.

**Table 3:** Type 1 correction factor for the major species of PC, SM and PE of milk.

PC Class	Correction Factor	SM Class	Correction Factor	PE Class	Correction Factor
IS	1.6223	IS	1.6196	IS	1.5686
PC 30:0	1.5689	SM 32:1	1.5486	PE 32:1	1.5511
PC 32:1	1.6042	SM 34:1	1.5837	PE 34:2	1.5860
PC 32:0	1.6045	SM 38:1	1.6563	PE 34:1	1.5863
PC 34:2	1.6403	SM 39:1	1.6750	PE 36:4	1.6214
PC 34:1	1.6406	SM 40:1	1.6938	PE 36:3	1.6217
PC 36:3	1.6771	SM 41:1	1.7129	PE 36:2	1.6220
PC 36:2	1.6774	SM 42:1	1.7322	PE 36:1	1.6223
PC 36:1	1.6777				
Ratio to IS	0.967–1.034		0.956–1.070		0.989–1.034

Note: Data extracted from LIPID MAPS Structure Database.

**Table 4:** Matrix effect (%) in relation to lipid extraction methods.

Lipids	Milk-11		Milk-12	
	One-Phase	Folch	One-Phase	Folch
PC	97 ± 0.3	90 ± 8.7	98 ± 1.5	89 ± 9.0
SM	117 ± 3.9	104 ± 8.4	117 ± 2.9	103 ± 10.5
PE	91 ± 2.9	78 ± 3.0	89 ± 5.8	80 ± 5.0
PS	109 ± 5.0	96 ± 8.3	107 ± 1.7	92 ± 3.9
PI	93 ± 5.0	79 ± 0.2	90 ± 2.4	85 ± 0.2

Note: The deuterated standards used were Mouse Splash Lipidomix standards (diluted by 32-fold). The standards were spiked into 40- or 44-fold diluted lipophilic and deproteinised matrix extracted by the Folch or one-phase method. LC method used was Hilic-4. Each value is the mean of two analyses (±SD).

**Table 5:** Relative proportion (%) of species within each class and sum concentration of each phospholipid class of milk (Milk-11) as determined by 3 different LC elution gradients.

Lipid Class and Species	Hilic-M4	Hilic-M5	Hilic-M6
PC 30:0	15.9 ± 0.11	15.4 ± 0.29	15.1 ± 0.29
PC 32:1	6.4 ± 0.12	6.1 ± 0.04	6.4 ± 0.17
PC 32:0	15.8 ± 0.26	15.1 ± 0.19	15.5 ± 0.44
PC 34:2	8.1 ± 0.10	8.2 ± 0.17	8.2 ± 0.22
PC 34:1	26.9 ± 0.18	27.1 ± 0.50	27.1 ± 0.47
PC 36:3	6.4 ± 0.12	6.6 ± 0.05	6.4 ± 0.10
PC 36:2	13.5 ± 0.19	14.0 ± 0.18	14.0 ± 0.25
PC 36:1	7.0 ± 0.21	7.5 ± 0.11	7.3 ± 0.13
PC sum conc (µg/mL)	71.8 ± 1.7	73.4 ± 1.1	73.0 ± 0.8
SM 34:1	28.4 ± 0.48	28.7 ± 1.05	28.8 ± 1.68
SM 38:1	9.0 ± 0.12	8.8 ± 0.47	8.9 ± 0.23
SM 39:1	13.7 ± 0.13	13.6 ± 0.27	13.6 ± 0.21
SM 40:1	21.6 ± 0.19	21.5 ± 0.13	21.6 ± 0.58
SM 41:1	16.5 ± 0.28	16.6 ± 0.24	16.4 ± 0.49
SM 42:1	10.7 ± 0.18	10.9 ± 0.21	10.6 ± 0.23
SM sum conc (µg/mL)	44.5 ± 0.7	46.3 ± 1.8	44.7 ± 0.3
PE 32:1	2.4 ± 0.24	2.3 ± 0.24	2.1 ± 0.13
PE 34:2	8.2 ± 0.27	8.5 ± 0.15	8.6 ± 0.53
PE 34:1	12.4 ± 0.27	12.6 ± 0.24	12.9 ± 0.79
PE 36:4	5.5 ± 0.34	5.2 ± 0.22	5.2 ± 0.43
PE 36:3	18.3 ± 0.80	17.6 ± 0.17	17.5 ± 0.21
PE 36:2	42.1 ± 2.19	42.8 ± 0.64	42.5 ± 0.77
PE 36:1	11.1 ± 0.52	11.1 ± 0.12	11.2 ± 0.16
PE sum conc (µg/mL)	83.9 ± 4.2	81.4 ± 1.7	80.1 ± 5.7
PS 34:1	5.0 ± 0.31	5.3 ± 0.13	5.1 ± 0.29
PS 36:3	7.9 ± 0.34	7.3 ± 0.41	7.5 ± 0.22
PS 36:2	29.4 ± 0.66	28.6 ± 0.24	29.6 ± 0.92
PS 36:1	39.5 ± 1.06	40.2 ± 0.55	37.9 ± 1.07
PS 38:5	5.1 ± 0.33	5.2 ± 0.39	5.5 ± 0.86
PS 38:4	6.9 ± 0.22	7.5 ± 0.31	8.4 ± 1.05
PS 40:5	6.2 ± 0.18	5.9 ± 0.14	5.9 ± 0.31
PS sum conc (µg/mL)	35.9 ± 2.1	36.1 ± 0.5	36.8 ± 0.5
PI 34:1	6.2 ± 1.27	8.3 ± 0.60	8.2 ± 0.69
PI 36:2	35.5 ± 0.32	33.9 ± 1.11	35.2 ± 1.64
PI 36:1	32.2 ± 1.30	30.0 ± 0.52	30.9 ± 1.11
PI 38:5	8.7 ± 0.54	9.5 ± 1.27	8.8 ± 0.49
PI 38:4	10.7 ± 0.04	11.3 ± 0.60	11.0 ± 1.10
PI 38:3	6.7 ± 0.17	7.1 ± 0.31	6.0 ± 0.35
PI sum conc (µg/mL)	20.0 ± 2.8	19.6 ± 1.6	20.7 ± 1.8

Note: LC separation was by the Kinetex Hilic column using three elution programmes. Lipids were extracted by the Folch method. Lipid concentration was measured using the one-point IS calibration method. Each value is the mean of 3 analyses (±SD). Sample dilution factor was 40.

**Table 6:** Comparison of phospholipid concentration (µg/mL) of milk (Milk-11) determined by RP-LC-MS and Hilic-MS.

Lipid Class and Species	Hilic-M4	C8
PC 30:0	11.4 ± 0.1	8.8 ± 0 *
PC 32:1	4.6 ± 0.1	4.4 ± 0.05
PC 32:0	11.3 ± 0.3	10.4 ± 0.27
PC 34:2	5.8 ± 0.1	5.3 ± 0.03
PC 34:1	19.3 ± 0.3	18.5 ± 0.46
PC 36:3	4.6 ± 0.2	3.7 ± 0.02 *
PC 36:2	9.7 ± 0.3	7.7 ± 0.05 *
PC 36:1	5.0 ± 0.3	4.3 ± 0.05 *
PC sum conc (µg/mL)	71.8 ± 1.7	63.2 ± 0.73 *
SM 34:1	12.6 ± 0.3	12.2 ± 0.1
SM 38:1	4.0 ± 0.1	4.6 ± 0.2 *
SM 39:1	6.1 ± 0.1	7.4 ± 0.2 *
SM 40:1	9.6 ± 0.1	16.3 ± 1.3 *
SM 41:1	7.3 ± 0.2	9.8 ± 0.5 *
SM 42:1	4.8 ± 0.1	5.9 ± 0.2 *
SM sum conc (µg/mL)	44.5 ± 0.7	56.0 ± 2.4 *
PE 32:1	2.0 ± 0.1	1.5 ± 0.07 *
PE 34:2	6.9 ± 0.6	6.7 ± 0.24
PE 34:1	10.4 ± 0.7	13.4 ± 0.24 *
PE 36:4	4.6 ± 0.5	3.5 ± 0.05 *
PE 36:3	15.3 ± 1.4	14.5 ± 0.31
PE 36:2	35.3 ± 1.5	43.7 ± 0.56 *
PE 36:1	9.3 ± 0.8	20.9 ± 0.05 *
PE sum conc (µg/mL)	83.9 ± 4.2	104.2 ± 1.28 *

Note: Milk lipids were extracted by the Folch method. The multistep gradient was used for C8 separation and Hilic-4 method for Hilic-MS. MS resolution was set to 240,000. Sample dilution factor was 40. Lipid concentration was measured using the one-point IS calibration method. Each value is the mean of 3 analyses (±SD). * Indicates significant difference (*p* < 0.05) by *t*-test.

## Data Availability

The data are avaliable from the corresponding author.

## Supplementary Materials

The following are available online at https://www.mdpi.com/article/10.3390/foods12010163/s1, Figure S1: Yield of phospholipids as influenced by the extraction methods. A: Milk-11; B: Milk-12. LC separation was by the Hilic-4 method. Each column is the mean value of 3 analyses. Sample dilution factor was 44-fold and 40-fold for the one-phase method and the Folch method, respectively. Lipid concentration was measured using the one-point IS calibration method. * Indicates significant difference by *t*-test (*p* < 0.05), Figure S2: Retention time of major PC, SM and PE species of milk vs. IS. Milk lipids were extracted by the Folch method. The multistep gradient programme was used for RP-LC-MS, Table S1: Detailed information on Mouse Splash^®^ Lipidomix standards (Avanti Lipids), Table S2: Elution programmes for Hilic-MS.
