# Supplementary material for: Comparison of Workflows for Milk Lipid Analysis: Phospholipids
Source: Foods. 2022 Dec 28;12(1):163. doi: 10.3390/foods12010163 (PMC9818897; doi:10.3390/foods12010163)
Supplement: Supplementary file 1 [file foods-12-00163-s001.zip › foods-2087926-supplementary.pdf]

Supplementary Materials

Table S1. Detailed information on Mouse Splash® Lipidomix standards (Avanti Lipids).

Compound Name	Molecular Weight	Exact Mass	Chemical Formula	Conc. (µg/mL)*	Conc. µM*
**15:0-18:1(d7) PC**	753.11	752.61	$C_{41}H_{73}D_7NO_8P$	**75.3**	**100**
**15:0-18:1(d7) PE**	711.03	710.56	$C_{38}H_{67}D_7NO_8P$	**5.0**	**7**
**15:0-18:1(d7) PS (Na Salt)**	777.02	776.53	$C_{39}H_{66}D_7NNaO_{10}P$	**15.5**	**20**
**15:0-18:1(d7) PG (Na Salt)**	764.02	763.54	$C_{39}H_{67}D_7NaO_{10}P$	**3.8**	**5**
**15:0-18:1(d7) PI (NH_4 Salt)**	847.13	846.60	$C_{42}H_{75}D_7NO_{13}P$	**16.9**	**20**
**15:0-18:1(d7) PA (Na Salt)**	689.94	689.50	$C_{36}H_{61}D_7NaO_8P$	**6.9**	**10**
**18:1(d7) Lyso PC**	528.72	528.39	$C_{26}H_{45}D_7NO_7P$	**23.8**	**45**
**18:1(d7) Lyso PE**	486.64	486.35	$C_{23}H_{39}D_7NO_7P$	**1.0**	**2**
**18:1(d7) Chol Ester**	658.16	657.64	$C_{45}H_{71}D_7O_2$	**164.5**	**250**
**C18(Plasm)-18:1(d9) PC**	781.19	780.67	$C_{44}H_{77}D_9NO_7P$	**15.6**	**20**
**15:0-18:1(d7) DAG**	587.98	587.55	$C_{36}H_{61}D_7O_5$	**8.8**	**15**
**15:0-18:1(d7)-15:0 TAG**	812.37	811.77	$C_{51}H_{89}D_7O_6$	**28.4**	**35**
**d18:1-18:1(d9) SM**	738.12	737.64	$C_{41}H_{72}D_9N_2O_6P$	**14.8**	**20**
**C18(Plasm)-18:1(d9) PE**	739.11	738.62	$C_{41}H_{71}D_9NO_7P$	**3.7**	**5**

*Concentrations are based on the isotopic purity of each individual compound

Table S2. Elution programmes for Hilic-MS.

Hilic-M4		Hilic-M5		Hilic-M6	
**Time (min)**	**A %**	**Time (min)**	**A %**	**Time (min)**	**A %**
0	5	0	5	0	5
8	30	8	20	14	20
9	50	9	50	15	50
13	50	13	50	19	50
14	5	14	5	20	5
20	5	20	5	26	5
20	End	20	End	26	End

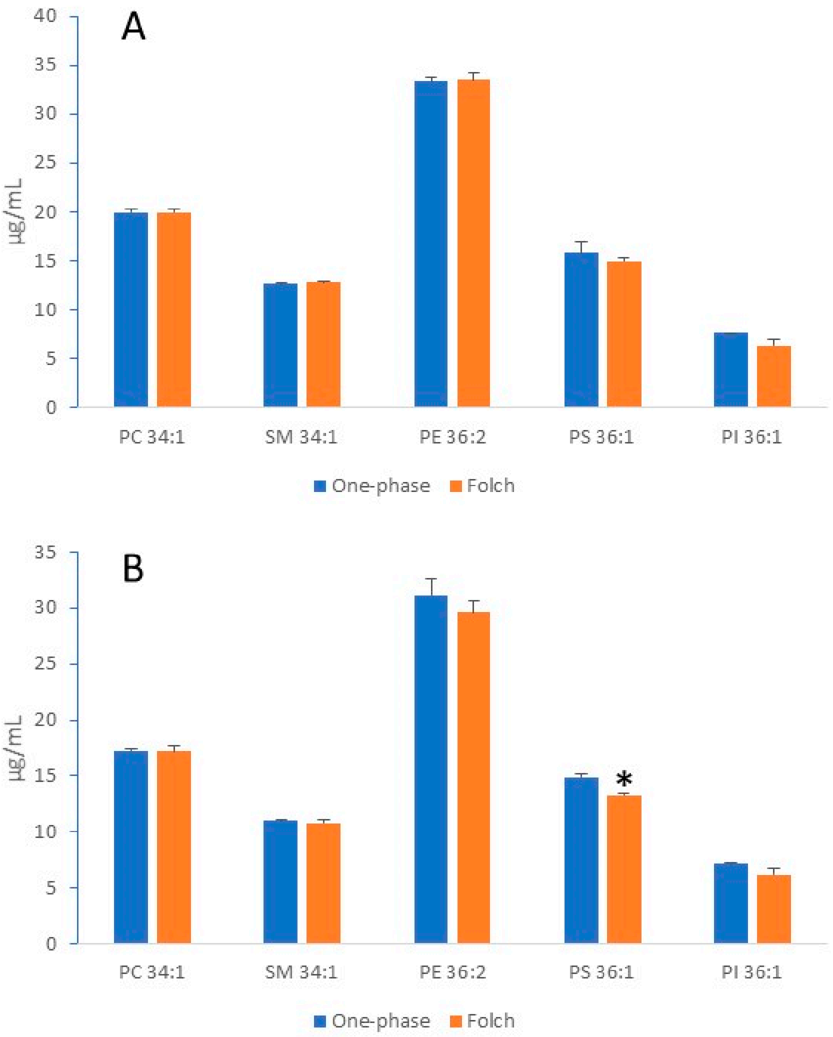

Figure S1. Yield of phospholipids as influenced by the extraction methods. A: Milk-11; B: Milk-12. LC separation was by the Hilic-4 method. Each column is the mean value of 3 analyses. Sample dilution factor was 44-fold and 40-fold for the one-phase method and the Folch method respectively. Lipid concentration was measured using the one-point IS calibration method. * Indicates significant difference by t-test ($p < 0.05$).

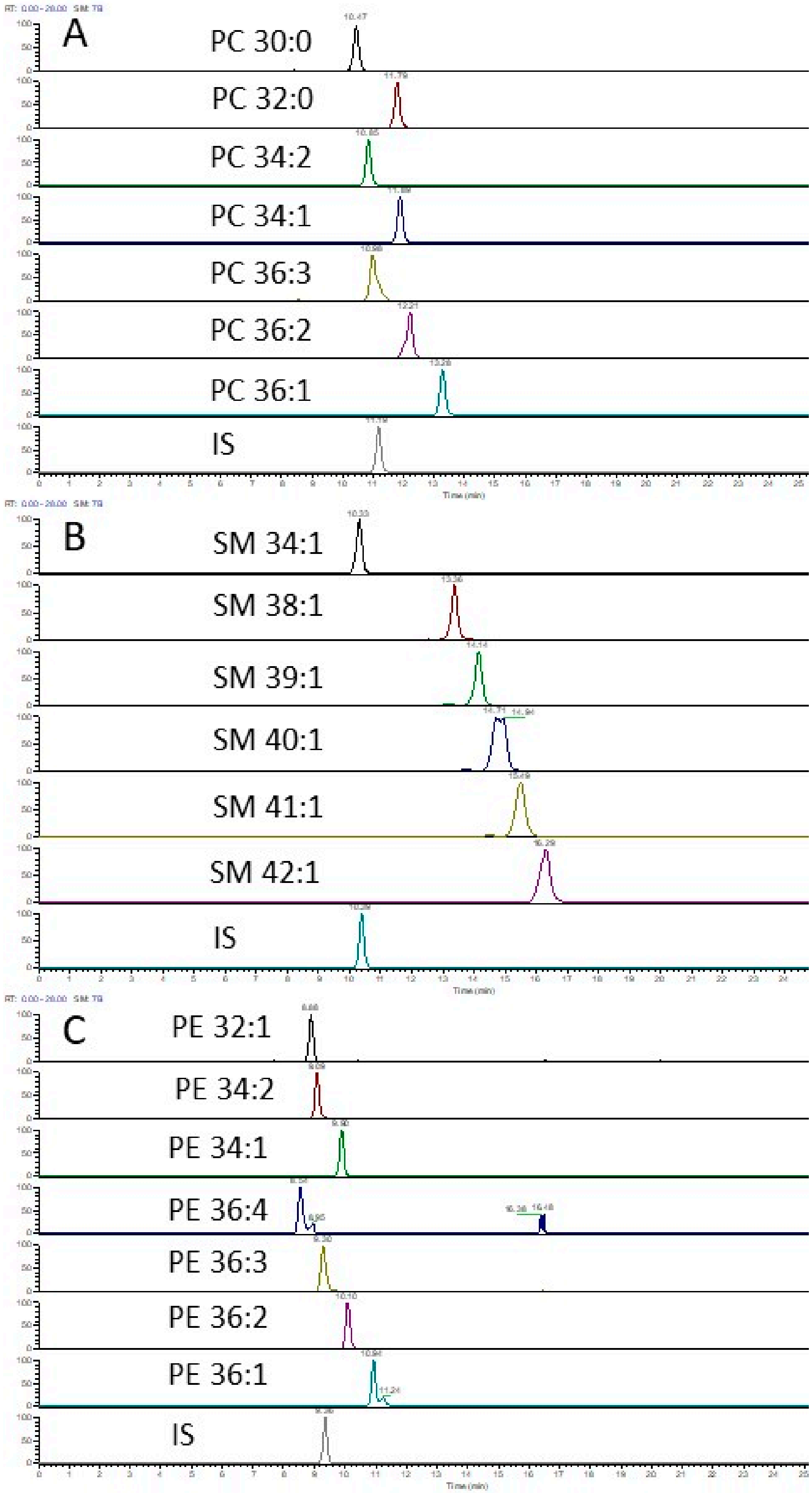

Figure S2. Retention time of major PC, SM and PE species of milk vs IS. Milk lipids were extracted by the Folch method. The multistep gradient programme was used for RP-LC-MS.
